# Disruption of the pleiotropic gene *scoC* causes transcriptomic and phenotypical changes in *Bacillus pumilus* BA06

**DOI:** 10.1186/s12864-019-5671-8

**Published:** 2019-04-30

**Authors:** Lin-Li Han, Yong-Cheng Liu, Cui-Cui Miao, Hong Feng

**Affiliations:** Key Laboratory for Bio-resources and Eco-Environment of the Ministry of Education, Sichuan Key Laboratory of Molecular Biology and Biotechnology, College of Life Sciences, Sichuan University, Chengdu, People’s Republic of China

**Keywords:** *Bacillus pumilus*, Flagella, Motility, Protease, *scoC*, Transcriptome

## Abstract

**Background:**

*Bacillus pumilus* is a Gram-positive and endospore-forming bacterium broadly existing in a variety of environmental niches. Because it produces and secrets many industrially useful enzymes, a lot of studies have been done to understand the underlying mechanisms. Among them, *scoC* was originally identified as a pleiotropic transcription factor negatively regulating protease production and sporulation in *B. subtilis*. Nevertheless, its role in *B. pumilus* largely remains unknown.

**Results:**

In this study we successfully disrupted *scoC* gene in *B. pumilus* BA06 and found increased total extracellular protease activity in *scoC* mutant strain. Surprisingly, we also found that *scoC* disruption reduced cell motility possibly by affecting flagella formation. To better understand the underlying mechanism, we performed transcriptome analysis with RNA sequencing. The result showed that more than one thousand genes were alternated at transcriptional level across multiple growth phases, and among them the largest number of differentially expressed genes (DEGs) were identified at the transition time point (12 h) between the exponential growth and the stationary growth phases. In accordance with the altered phenotype, many protease genes especially the *aprE* gene encoding alkaline protease were transcriptionally regulated. In contrast to the finding in *B. subtilis*, the *aprN* gene encoding neutral protease was transcriptionally downregulated in *B. pumilus*, implicating that *scoC* plays strain-specific roles.

**Conclusions:**

The pleiotropic transcription factor ScoC plays multiple roles in various cellular processes in *B. pumilus,* some of which were previously reported in *B. subtilis*. The supervising finding is the identification of ScoC as a positive regulator for flagella formation and bacterial motility. Our transcriptome data may provide hints to understand the underlying mechanism.

**Electronic supplementary material:**

The online version of this article (10.1186/s12864-019-5671-8) contains supplementary material, which is available to authorized users.

## Background

*Bacillus pumilus* is a Gram-positive bacterium with great potential for industrial applications. It produces and secretes a variety of useful enzymes [1–5]. It has also been developed as bio-pesticide or bio-fertilizer [6, 7]. Moreover, because of its favorable growth and secretion features, *B. pumilus* has been engineered as host organism for recombinant protein production [8–10].

Previously, we isolated a *B. pumilus* strain named BA06 for its high level production of extracellular alkaline protease, which could be used for leather processing [11–14]. To further elevate the protease production, better knowledge regarding how it is regulated would be necessary. We are interested in the gene *scoC* (also called as *hpr*) because it has been shown to be a pleiotropic regulator in *B. subtilis* and its homolog also exits in *B. pumilus* [15]. *scoC* was first recognized in the study of *B. subtilis* mutants which could over-produce alkaline and neutral proteases [16] and showed a glucose-insensitive sporulation phenotype [17]. Later, *scoC* was cloned [18] and its recombinant protein could bind a consensus DNA element (5′-RATANTATY-3′) that lies upstream of the *aprE* (encoding alkaline protease) and *nprE* (also called as *aprN*, encoding neutral protease), which acted as a negative regulator of transcription of *aprE* and *nprE* [19]. In addition, ScoC was also demonstrated to regulate multiple target genes involved in a broad range of biological processes such as the signaling peptide transport systems, *app* and *opp* [20] and bacilysin production in *B. subtilis* [21]. Furthermore, the transcriptome analysis showed that *scoC* regulated expression of more than 560 genes in *B. subtilis,* directly or indirectly [22].

Although we found *scoC* expression was peaked at the exponential-growing cells and significantly declined in the later growing phases [23], its exact role in *B. pumilus* remains unknown. In this work, we disrupted the *scoC* gene with homologous recombination. The *scoC* mutant strain showed increased extracellular protease activity and decreased bacterial motility possible due to reduced flagellar formation. These phenotypes are consistent with the transcriptome profiling changes with upregulation or downregulation of the related genes.

## Results

### Phenotypical characterization of *scoC* deletion mutant

**Fig. 1:**
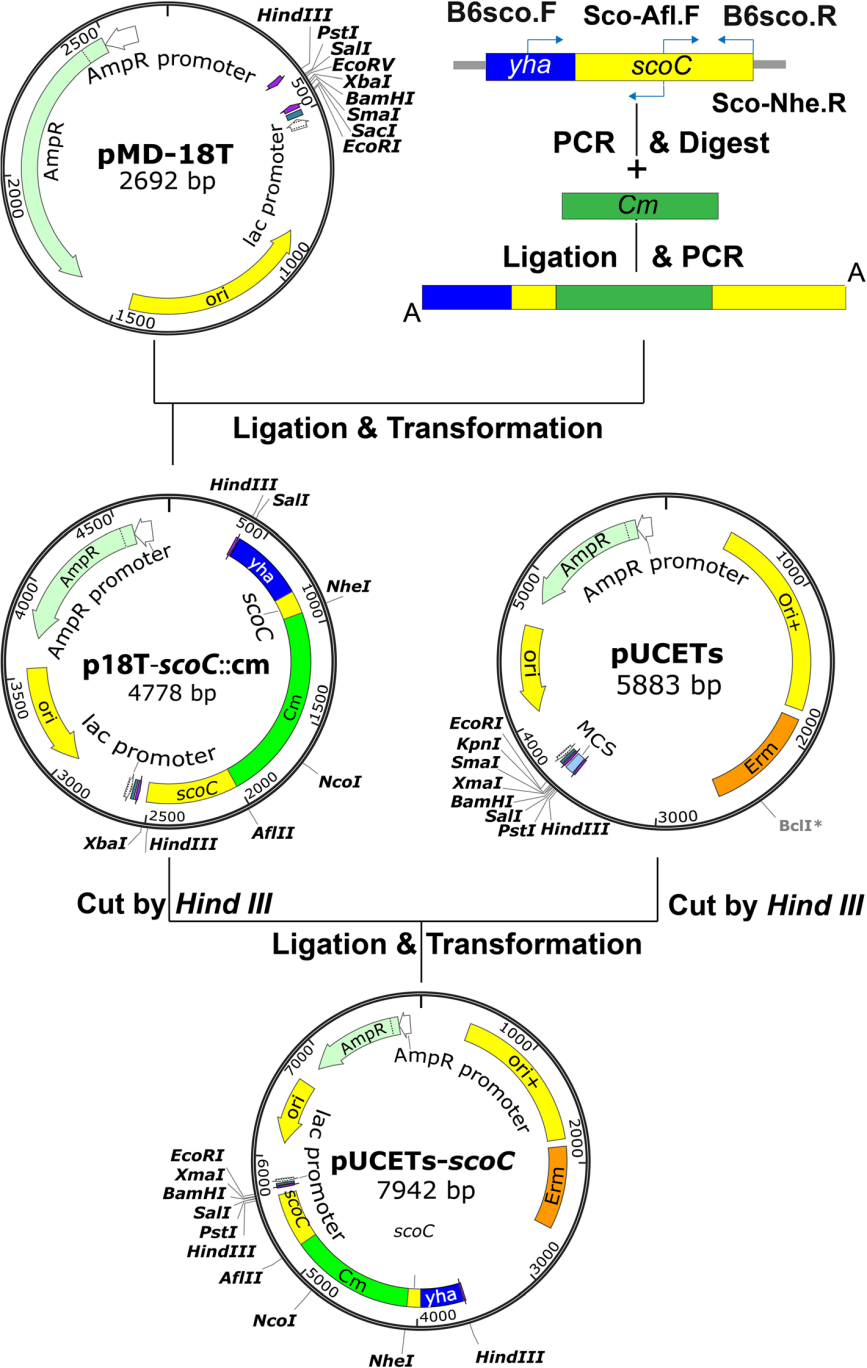
Outline for construction of the disrupting vector pUCETs-*scoC*::cm

A *scoC* deletion mutant strain (BA06-∆*scoC*) was successfully disrupted by homologous recombination following the strategy as showed in Fig.1 and confirming by colony PCR and DNA sequencing (Additional file 1: Figure S1). Furthermore, an overexpression strain (∆*scoC*/*scoC*^+^) was also constructed to overexpression ScoC on the multiple-copies plasmids (pSU03-*scoC*).

**Fig. 2:**
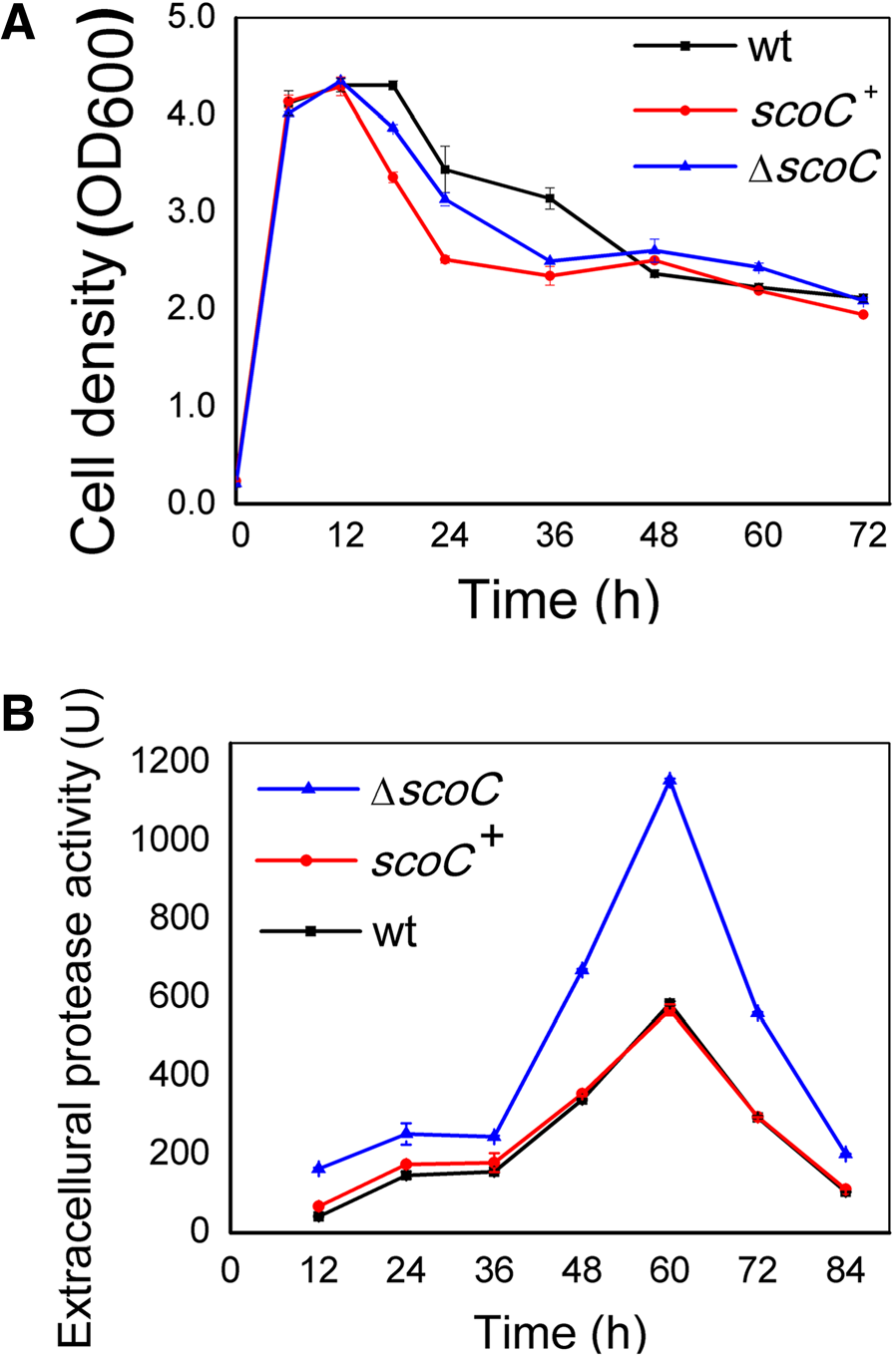
Comparison of the growth pattern (**a**) and extracellular proteolytic activity (**b**) of the *scoC* mutant (∆*scoC*), *scoC* overexpression (*scoC*^+^), and the wild-type (wt) strains of *B. pumilus* BA06. The bacterial strains were grown in the minimal medium at 37 °C with shaking at 200 rpm

Firstly, we compared the growth pattern of the wild-type (wt), *scoC* deletion mutant (BA06-∆*scoC*), and *scoC*/*scoC*^+^ overexpression strains. Figure 2a showed that the defect or overexpression of *scoC* did not affect the cell growth in MM broth in comparison with the wt. Secondly, since the previous studies in *B. subtilis* showed that ScoC negatively regulated protease genes of *aprE* and *nprE*, and *scoC* disruption led to significant increase of extracellular protease activity [19, 24], the total extracellular protease activity was determined in of *B. pumilus* (Fig. 2b). It was showed that *scoC* deletion caused 2-fold increase in extracellular proteolytic activity compared to the wt at the time point of 60 h. On the other hand, overexpression of *scoC* in the ∆*scoC*/*scoC*^+^ strain reversed to diminish the increased extracellular protease activity. Like in *B. subtilis*, ScoC acted as a negative regulator of extracellular proteases in *B. pumilus*.

**Fig. 3:**
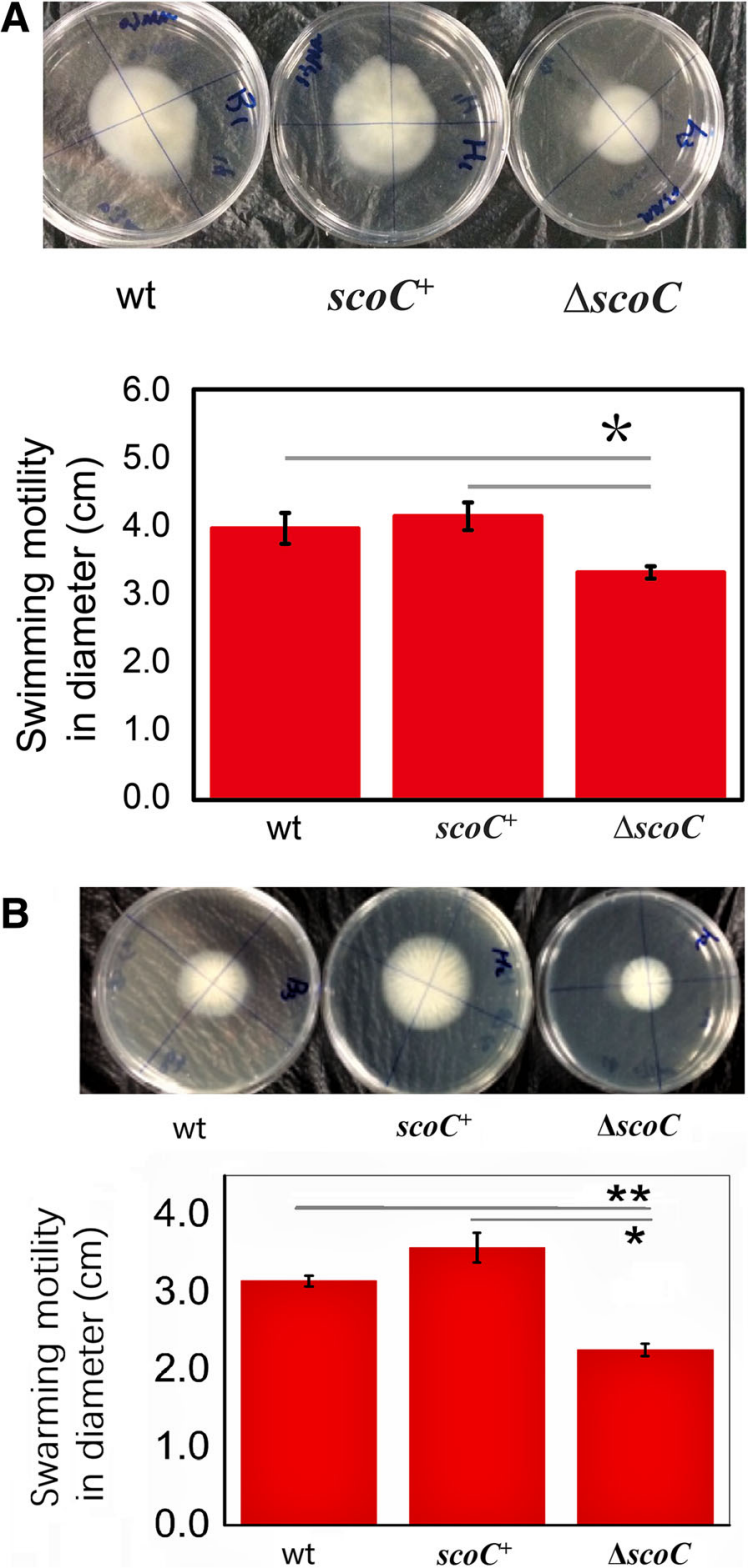
Analysis of cell motility of *scoC* mutant (∆*scoC*), *scoC* overexpress (*scoC*^+^), and the wild-type (wt) strains of *B. pumilus* BA06. Swimming motility (**a**) was assayed on the minimal medium with 0.3% agar. Swarming mobility (**b**) was assayed on LB plate with 0.7% agar. The colony expanding diameter was measured and used for AOVA with *p* < 0.05 (*) or 0.01 (**)

Next, we examined the bacterial motility on the solid and semi-solid plates with agar concentrations of 0.7 and 0.3% [25]. Figure 3 showed that the colony size of BA06-∆*scoC* was much smaller than that of the wt and ∆*scoC*/*scoC*^+^ strains on LB plate with 0.7% agar and MM plate with 0.3% agar, respectively, indicating that both the swarming and swimming motility were compromised after *scoC* disruption. The colony expansion in diameter of BA06-∆*scoC* was statistically different from the wt and overexpression strains with *p* < 0.05 and 0.01, respectively. These results indicated that *scoC* could modulate the cell motility of *B. pumilus*.

**Fig. 4:**
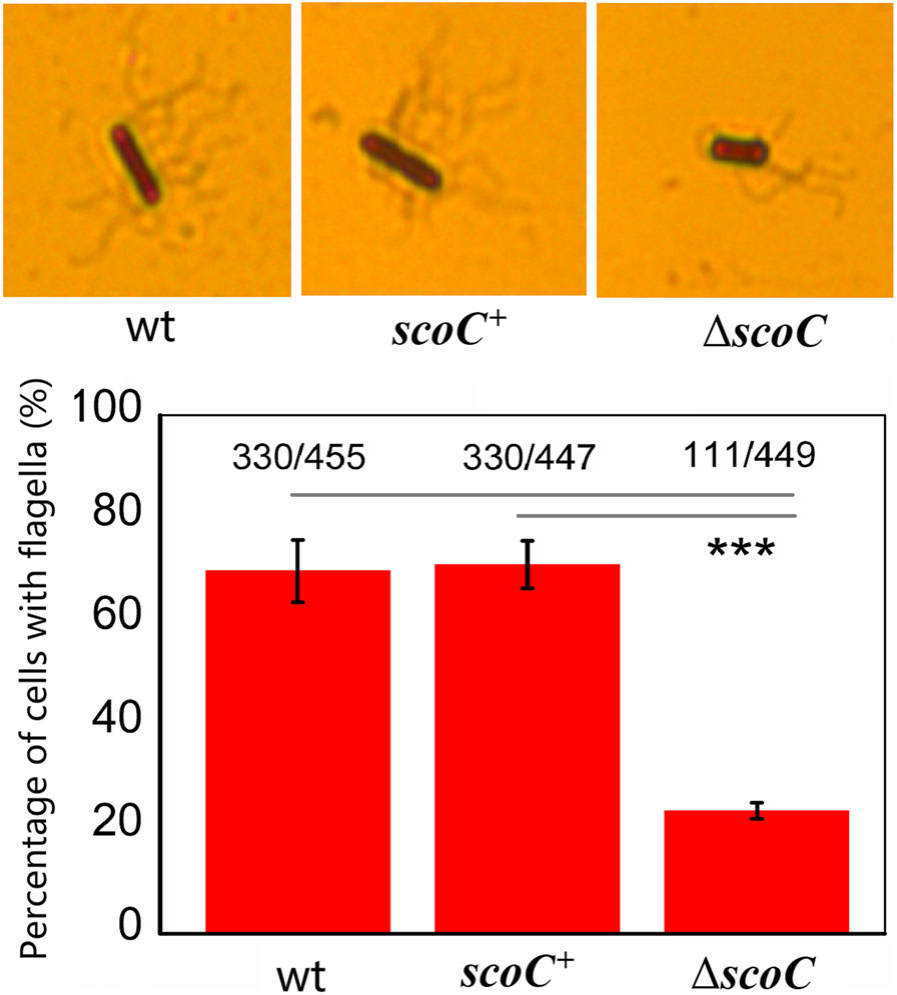
Analysis of flagellar biosynthesis. The number of cells with flagella (> 4 per cell) and without flagella (< = 3) were counted under oil microscope. Percentage of the cells with flagella were used for AOVA with *p* < 0.001 (***)

Since flagellum is the movement organ of bacteria, we counted the cell number with flagella or without flagella for the three *B. pumilus* strains. It was found that *scoC* disruption reduced the flagella formation (Fig. 4). The percentage of cells with flagella were only 23.7% for BA06-∆*ScoC*. By contrast, the percentages were 69.8 and 71.1% for the wt and overexpression strains, respectively. Of interest, previous studies done in *B. subtilis* showed that many flagellar genes were downregulated in *scoC* deletion mutant [22]. These results suggested that decrease of cell motility of BA06-∆*scoC* was at least partially resulted from the reduction of flagella formation.

In addition, formation of endospore and biofilm was also examined. In contrast to *B. subtilis*, no significant difference was found among the three *B. pumilus* stains (Additional file 2: Figure S2), implicating ScoC also plays strain-specific role.

### Transcriptome profiling of *scoC* mutant

**Fig. 5:**
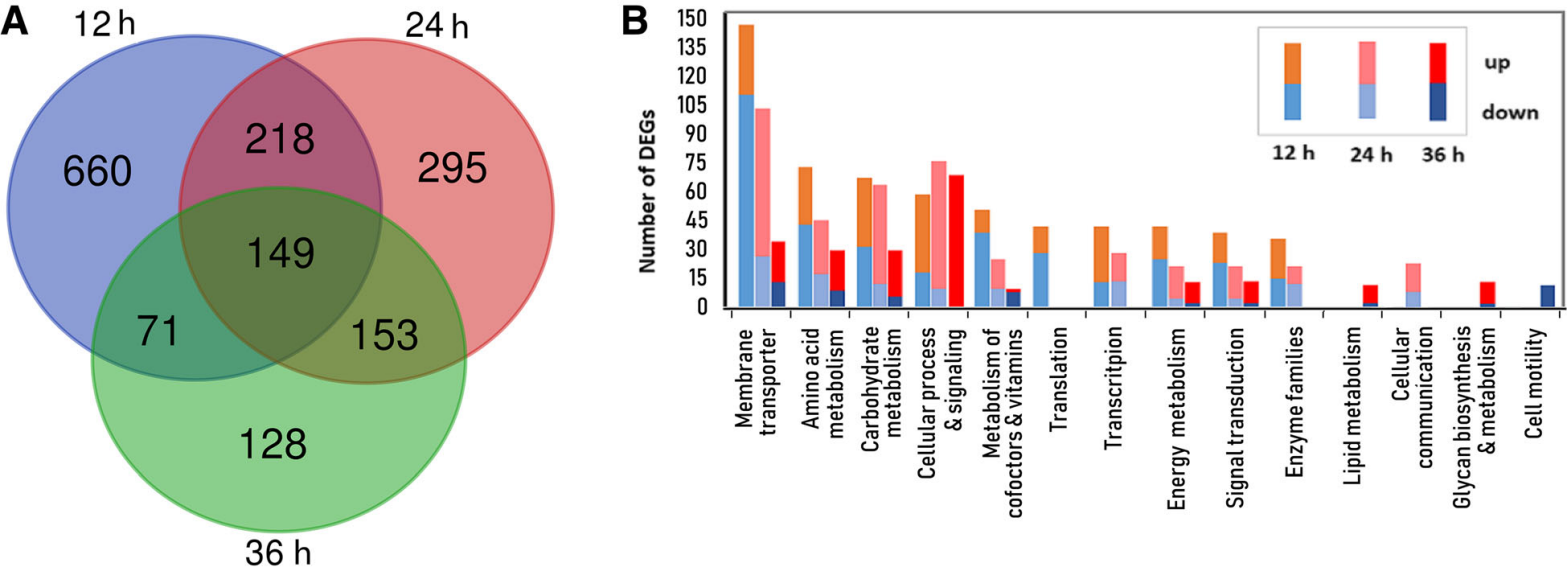
Venn diagram analysis (**a**) and KEGG enrichment (**b**) of the differentially expressed genes (DEGs) between *scoC* mutant and the wild-type strain of *B. pumilus* BA06 across three time-points

To better understand the mechanism underlying the observed phenotypes in *B. pumilus*, we performed comparative transcriptome profiling using RNA sequencing. Overall, a total 1674 differentially expressed genes (DEGs) with the expression level > 2.5-fold change and *p* < 0.01 were identified across the three time points at which the BA06-∆*scoC* and wt cells were compared (Fig. 5a, Additional file 3: Table S1). The most DEGs were identified at the time point of 12 h with 531 up-regulated and 467 down-regulated. And DEGs subsequently declined at the later growth phases, suggesting that *scoC* may play a major role at the transition phase.

By KEGG pathway analysis, the DEGs were categorized into various metabolic pathways, of which the top 10 were displayed in Fig. 5b. Overall, the expression patterns are very similar between *B. subtilis* and *B. pumilus* after *scoC* disruption. The largest group with altered transcriptional level in the *scoC* deletion mutant belongs to the KEGG category of membrane transport [22]. For example, some putative operons involved in the metal ion transport systems (cds3101–3105; cds3029–3032) were downregulated in BA06-∆*scoC* strain (Additional file 3: Table S1). In *B. subtilis*, some operons like *opp* and *app* encoding oligopeptide transport systems have been experimentally confirmed to be directly regulated by *scoC* [20]. In addition, many affected genes were also enriched in amino acid metabolism, carbohydrate metabolism, translation, transcription, etc.

Nevertheless, there are also significant differences from *B. subtilis*. For instance, two protease genes *aprE* (cds0935) and *aprX* (cds1594) showed same expression pattern, i.e. upregulation in both strains at 24 and 36 h (Table 1), while the neutral protease gene *aprN* (cds2061) was decreased in *B. pumilus* but increased in *B. subtilis*, respectively. Furthermore, the other protease gene like *epr* (cds0246) and *vpr* (cds3474) did not changed obviously in transcription level in *B. pumilus* after *scoC* deletion (Table 1). Because the proteases like AprE is regulated by both positive and negative regulators in *B. subtilis*, herein we examined transcriptional level of the homologous regulatory genes in *B. pumilus*. It was showed that the negative regulator gene *abrB* (cds0014) and *sinR* (cds2171) did not change (Additional file 3: Table S1). However, the sensor histidine kinase gene *degS* (cds3226) were upregulated at 12 h, which may promote the phosphorylation level of DegU and in turn enhance the transcription of *aprE* [26]. This is consistent with increased *aprE* expression.

**Table 1:** Relative expression level (TPM) of the protease genes of the *scoC* mutant (∆*scoC*) and the wild-type (wt) strain of *B. pumilus* BA06 across three time-points

Gene ID	Gene	Protein	*B. pumilus* (wt)			∆*scoC* mutant		
			12 h	24 h	36 h	12 h	24 h	36 h
CDS0232	*wprA*	Cell wall-associated protease	121	72	127	120	140	144
CDS0246	*epr*	Minor extracellular protease	58	50	47	45	40	33
CDS0935	*aprE*	Subtilisin E	10,201	5730	11,622	27,522^a^	53,512^a^	33,455^a^
CDS1190	*isp*	Major intracellular protease	10,281	2299	6415	3490^a^	5368^a^	8946
CDS1401	*bpr*	Bacillopeptidase F	439	233	597	282	499^a^	353
CDS1594	*aprX*	Serine protease AprX	386	16	55	24^a^	388^a^	1002^a^
CDS2061	*aprN*	Subtilisin NAT	274	188	292	61^a^	42^a^	51^a^
CDS3474	*vpr*	Minor extracellular protease	390	465	484	297	218	199

^a^indicating the significant fold-change with |log(FC)| > 1.3219 and p < 0.01

**Fig. 6:**
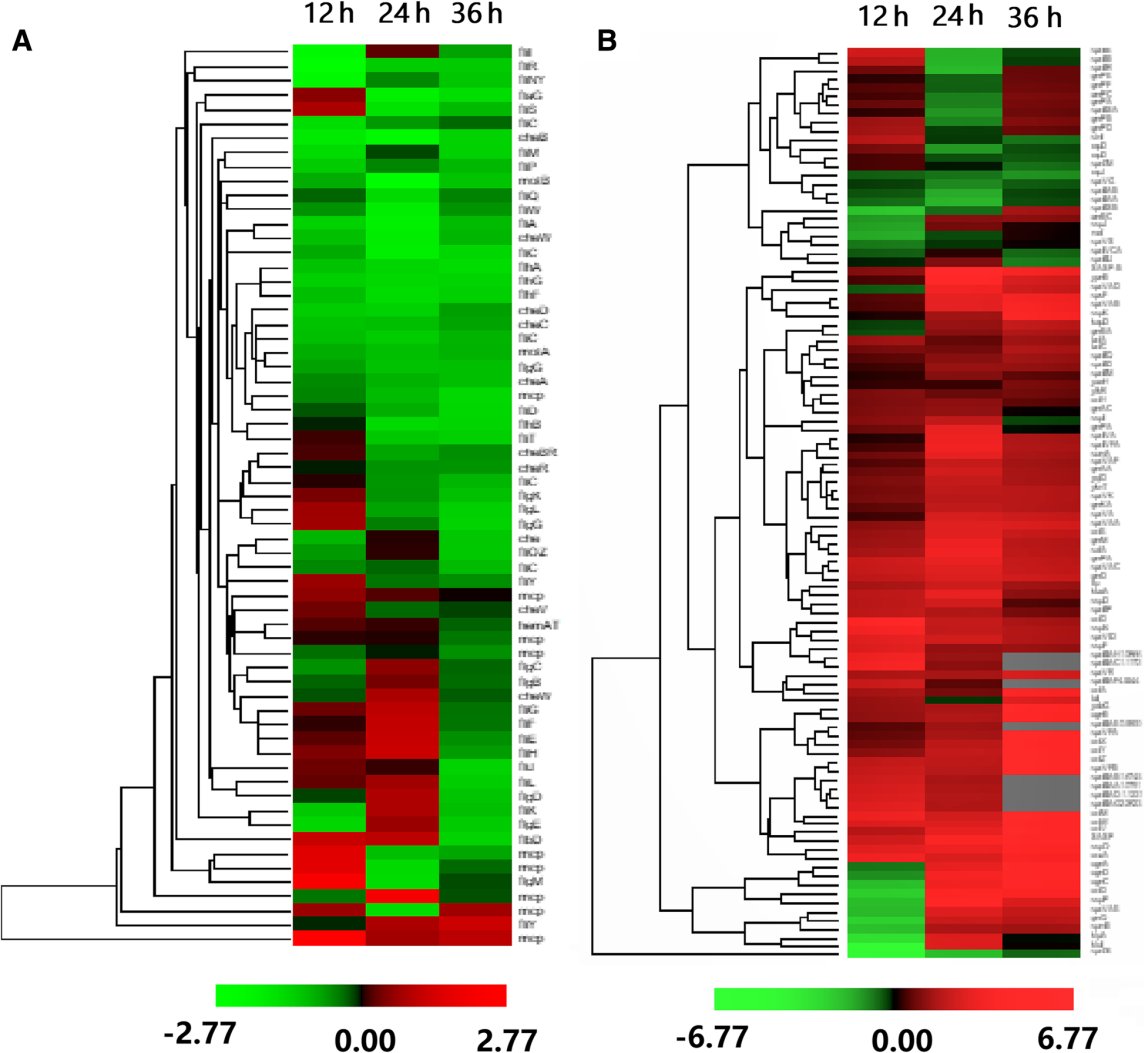
The matrix hierarchical cluster analysis of the differentially expressed genes categorized into the cell motility (**a**) and sporulation (**b**) between *scoC* mutant (∆*scoC*) and the wild-type (wt) strain of *B. pumilus* BA06 across three time points

Since the *scoC* mutation displayed defective flagella formation (Fig. 4), we examined the transcriptional profiling of flagella-related genes. In *B. pumilus*, these genes are organized as several operons (like *flc/che* and *motAB*) or individually scattered in the genome (Additional file 4: Figure S3). Overall, 63 genes categorized into the KEGG Bacterial Motility (k02035) and Flagellar Assembly (k02040) pathways have a trend to downregulation in BA06-∆*scoC* strain (Fig. 6a, Additional file 3: Table S1). Nevertheless, seldom genes were upregulated. For example, the *flgM* gene (cds3219, encoding an anti-sigma factor) were upregulated for about 3-fold at 12 h (Additional file 4: Figure S3). In the *fla/che* operon, the upregulated genes (*fliEFGH, fliJ*) may be transcribed from another independent promoter [27]. In addition, the gene *swrA* encoding a master regulator of motility that activates the cell motility in *B. subtilis* [28], was also upregulated in *scoC* deletion mutant of *B. pumilus*. Taken together, the transcriptome data suggest that the compromised flagella formation is at least in part due to downregulation of the flagellar structural genes after *scoC* deletion.

Another group of DEGs is enriched into the KEGG Cell Growth pathway (99978). Most of these genes related to sporulation were continually upregulated at all three time points in BA06-∆scoC (Fig. 6b), consistent with the finding that *scoC* disruption promoted sporulation in *B. subtilis* [17, 24]. However, no overt enhanced sporulation was observed in *B. pumilus* (Additional file 2: Figure S2A), implicating there might existed additional regulatory pathway in *B. pumilus*. Of note, these sporulation-related genes are still not found to interact directly with ScoC. Therefore, *scoC* may undergo an indirect pathway to regulate sporulation. For example, *scoC* can suppress sporulation by acting as a repressor of the signaling peptide transport systems, *opp* and *app* in *B. subtilis* [20]. Indeed, we found the *opp* operon (*opp*ABCDF, cds1038–1042) was downregulated at 12 h and then upregulated at 24 h in *B. pumilus scoC* mutant (Additional file 3: Table S1).

Lastly, to confirm the accuracy and reproducibility of the transcriptome data, 13 genes were selected for qPCR validation. RNA samples from the same cultures of MM at different growth phases were used as template. The data shown in Table S2 (Additional file 5) indicated that RNA-seq data were almost consistent with the qPCR results.

## Discussion

Previous studies have demonstrated that *scoC* negatively regulated a plethora of genes or operons in *B. subtilis* through direct binding to a consensus DNA element located in promoter region of these genes [16–20]. ScoC homologs have been found in many *Bacillus* species other than *B. subtilis*, but their functions remained largely unknown. In this work, we showed that disruption of *scoC* gene in *B. pumilus* increased extracellular protease activity and decreased cell motility, which could be attributed to transcriptional alternation of ScoC target genes.

Our data indicated *scoC* disruption leads to many genes to alter the transcriptional level across different growth phases. The large number of DEGs up to 1098 were enriched at 12 h, the transition point between exponential growth and stationary phase [23]. Since ScoC is recognized as one of the transition-state regulators that silence expression of genes involved in secondary metabolisms at the exponential growth phase [29]. By KEGG analysis, many genes categorized into the secondary metabolisms were identified to be upregulated, such as sporulation related genes and various enzymes after *scoC* disruption. On contrast, some genes involved in primary metabolisms like membrane transport and amino acid metabolism were also down-regulated (Fig. 5b). Therefore, we conclude that *scoC* may play a leading role in transcriptional regulation at the metabolic transition time.

*B. pumilus* encodes several extracellular proteases, of which AprE is the major one accounting for more than 70% of the total extracellular proteolytic activity [30]. In this work, we found the total extracellular proteolytic activity of the *scoC* mutant was significantly increased, which could be ascribed to the increase of *aprE* transcription. *aprE* has been showed as a direct target by ScoC [30], which is also regulated by multiple transcription factors such as AbrB, DegU/DegS, SinR, and Spo0A [31]. In viewing of our transcriptome data, DegS and Spo0A may partially contribute to increased transcription of *aprE* in the BA06-∆*scoC* strain. Unexpected, *aprN* encoding neutral protease was seriously downregulated in *scoC* deletion mutant (Table 1). That is totally different from *B. subtilis*, in which *aprN* was a direct target of ScoC [16, 19]. Therefore, *scoC* may underly additional mechanism to regulate expression of *aprN*. In addition, the transcription level of *epr* and *vpr* did not change significantly (Table 1). However, *epr* was reported to be only co-repressed by ScoC and SinR and individual mutant of *scoC* or *sinR* did not repress *epr*’s expression in *B. subtilis* [32]. The expression of *vpr* was not mediated by ScoC in *B. subtilis* [33]. Conclusively, these protease-encoding genes may be differentially regulated by ScoC in *B. pumilus*.

Another phenotypical change caused in *scoC* mutant is decrease of cell motility (Fig. 3) and defective flagella formation (Fig. 4). As expected, many of these genes involved in flagellar biosynthesis were indeed down-regulated (Fig. 6a). The similar observation was also found in in *B. subtilis* [22]. In *B. cereus*, the flagella-associated genes were upregulated in the swarming cells in comparison with the non-swarming cells [34]. All the experimental evidences indicate that the flagellar biosynthesis as well as cell mobility is associated with expression level of the relative genes. In *B. subtilis*, the studies on regulatory mechanism of flagellar biosynthesis are focusing on the *fla/che* operon, which containing 32 genes that encode the flagellar basal-body rod proteins. A SwrA protein has been reported as a master regulator in regulation of flagellar biosynthesis [28]. The gain-of function mutations in *swrA* increased the proportion of motile cells and the flagellar number per cells [25, 35]. Although our data showed that *swrA* (cds3194) was upregulated by about 3-fold at 12 h (Additional file 3: Table S1), the flagella formation was comprised (Fig. 4). A reasonable explain of this discrepancy is existence of additional regulators. For example, unphosphorylated DegU could bind an inverted repeat-like upstream of the *fla/che* promoter and then activated transcription; while the phosphorylated DegU played a contrast role to depress its expression by binding to another inverted repeat-like sequence in *fla/che* operon [36, 37]. In addition, SwrA activation of the *fla/che* expression may require DegU [38, 39], which may be compensated by enhancement of phosphorylation DegU by DegS because *degS* was up-regulated in transcription in *B. pumilus scoC* mutant.

In addition, ScoC may regulate flagellar biosynthesis through SigD-mediated pathway. Almost all the flagella-related genes belong to SigD regulon [40]. The gene *flgM* to encode anti-sigma factor against sigD was positively regulated by ScoC via binding to its promoter in *B. subtilis* [41, 42]. Our data showed that *flgM* (cds3219) was really upregulated by more than 4-fold at 12 h (Additional file 3: Table S1). Taken together, ScoC may modulate the flagellar biosynthesis through direct or indirect pathways in *B. pumilus*.

For *Bacillus*, it is an important to produce endospores under stress conditions. The formation of endospores begins with a period of stable growth of nutrient-poor and is restricted by advanced hierarchical regulation [43]. Spo0A is the master regulator to initiate endospore formation [44]. According to earlier studies, ScoC is a negative regulator for sporulation of *B. subtilis* [17, 18]. Our transcriptome data demonstrated that many sporulation genes were upregulated after *scoC* deletion (Fig. 6b). We did not observe any significant change of sporulation between the wt and *scoC* mutation strains. Since no target gene involved in sporulation has been identified [40], regulatory role of ScoC on sporulation remains unknown in *B. pumilus*.

## Conclusions

In conclusion, we found that disruption of *scoC* gene in *B. pumilus* caused increased extracellular protease activity, decreased motility and compromised flagella formation. All these features are well correlated with the transcriptional changes of the corresponding genes. However, our transcriptome data provides new hints to further investigate the putative target genes of ScoC in *B. pumilus*.

## Materials and methods

### Bacterial strains, plasmids and culture conditions

The bacterial strains used here are present in Table 2. The primers used to construct each plasmid are listed Table S3 (Additional file 6). All strains were grown in Luria-Bertani (LB) broth at 37 °C unless stated otherwise. When appropriate, LB broth was supplemented with 50 μg/mL kanamycin (Km) or 100 μg/mL ampicillin (Amp) for *Escherichia coli*, 5 μg/mL erythromycin (Erm) or 10 μg/mL chloramphenicol (Cm) for *B. pumilus*.

**Table 2:** The bacterial strains and plasmids used in this work

Strains and plasmids	Description	Reference
Strains		
*Escherichia coli* DH5α	*sup*E44Δ*lac*U169 (_Φ_80 *lacZ*ΔM15) *hds*R17 *rec*A1 *end*A1 *gyr*A96 *thi*-1 *rel*A1	Lab stock
*Bacillus pumilus* BA06	The wild-type strain	Lab stock
*B. pumilus* BA06-∆*scoC*	∆*scoC*; *Cm*	This work
*B. pumilus* (∆*scoC/scoC*^+^)	*scoC*^+^; Kan	This work
Plasmids		
pMD-18 T	T-vector; Amp; high copies	TaKaRa Co.
pHCMC02	Cm; high copies	[45]
pUCETs	Shuttling vector (*E. coli* and *Bacillus)*; temperature-sensitive *ori*; Erm; low copies	[46]
pSU03-Ap	expression vector (*Bacillus*); high copies	[47]
p18T-*scoC*::cm	*scoC*::Cm; Amp; high copies	This work
pUCETs:*scoC*::cm	*scoC*::Cm; Erm; low copies	This work
pSU03-*scoC*	*scoC;* promoter of GAPD; Kan; high copies	This work

All the restriction enzymes were purchased from Thermo Fisher Scientific Inc. (Waltham, MA, USA). T4 DNA ligase, *Pfu* DNA polymerase, Taq DNA polymerase, RTase and the Genome DNA Eraser Kit were obtained from TaKaRa Bio. Co. (Dalian, China). 2 × Real Time PCR EasyTM -SYBR Green I kit was purchased from Chengdu Fuji Biotechnology Co., Ltd. (Chengdu, China). TRIzol reagent was purchased from Invitrogen Co. (Carlsbad, CA, USA). Gel Recovery Kit and PCR Cycle recovery Kit was obtained from Omega Bio-tek, Inc. (Norcross, GA, USA).

### Construction of vectors

To construct the disruption vector, two DNA fragments covering the full *scoC* gene as well as its flanked sequences was amplified by *Pfu* DNA polymerase with primers (B6sco.F/Sco-Afl.F, and Sco-Nhe/B6sco.R) using genomic DNA of *B. pumilus* BA06 as template. The PCR fragments were purified and digested with *Afl* II and *Nhe* I, respectively. A 1095-bp *Afl* II-*Nhe* I fragment containing the chloramphenicol resistance gene (Cm) was obtained to digest the plasmid pHCMC02 [45]. Then, the Cm fragment was mixed and ligated with the above two PCR fragments using T4 DNA ligase. The ligated product was amplified using primers (B6sco.F and B6sco.R) and *Taq* DNA polymerase. The expected PCR product was directly cloned into pMD-18 T (Takara, Dalian, China) by A/T cloning using T4 DNA ligase, resulting plasmid p18T-*scoC*::cm. After confirmed by DNA sequencing, the *scoC*::cm fragment was obtained by digested with *Hin*d III and inserted into *Hin*d III-digested pUCETs [46] using T4 DNA ligase. The resulted vector pUCETs-*scoC*::Cm was used to disrupt the *scoC* gene.

The vector pSU03-*scoC* was constructed to overexpress *scoC* in *B. pumilus*. The promoter of the gene encoding glyceraldehyde-3-phosphate dehydrogenase (GAPDH) and the coding sequence of *scoC* were amplified using the primers (B06Pgpd.F/B06Pgpd.R, B06P-scoC.F/B06-scoC.R), respectively. And the two PCR fragments were recombined by overlapped PCR with the primers (B06Pgpd.F/B06-scoC.R). The resulting DNA fragment was digested with *Nde* I and *Xho* I. The vector sequence of pSU03-AP [47] was amplified by PCR using *Pfu* DNA polymerase and primers (pSU03ΔAp.F/pSU03ΔAp.R) and digested with *Nde* I and *Xho* I. Then, the above two fragments were ligated with T4 DNA ligase. The resulting plasmid was assigned as pSU03-*scoC*. A His6-tag was added at the 5′-upstream of *scoC*. The inserted sequence was confirmed by DNA sequencing.

During the vector construction, transformation of *E. coli* cells was performed as described by Sambrook et al. [48].

### Electroporation in *B. pumilus* BA06

The transformation of *B. pumilus* BA06 was referred to the high-osmolarity electroporation protocol in *B. subtilis* [49] with some modification. Briefly, the competent cells of *B. pumilus* BA06 were prepared as following: 0.5 mL of overnight culture in LB broth was transferred into 50 mL of LB broth (containing 0.5 M sorbitol and 5% betaine) and incubated at 37 °C up to OD600 to about 1.0; the cells were harvested by centrifugation at 5000 rpm and 4 °C for 10 min; after three washes with 50 mL of ice-cold washing buffer (0.5 M sorbitol, 0.5 M mannitol, 10% glycerol and 7.5% betaine), the cells were re-suspended in 1 mL electroporation buffer (0.5 M sorbitol, 1 M mannitol, 10% Glycerol and 7.5% betaine).

In the electroporation trials, 80 μL competent cells were mixed with 1–5 μL DNA (0.1–0.5 μg). And then, the mixture was transferred into an electroporation cuvette (0.1- cm electrode gap). The cells were exposed to a single electrical pulse (2400 V, 25 μF, 200 Ω) using Bio-Rad MicroPulser (Bio-Rad, USA). Immediately following the electrical discharge, 1 mL recovery medium (LB containing 0.5 M sorbitol and 0.38 M mannitol) were added to the cells. After incubating at 37 °C with vigor shaking for 3 h or longer, the cells were plated on LB-agar plates with appropriate antibiotic. Following the above protocol, dozens or up to hundreds of colonies per microgram plasmid DNA could be formed dependent upon various plasmids.

### Screening of *scoC* deletion mutant

The *B. pumilus* transformant hosting the vector pUCETs-*scoC*::Cm was inoculated in 2~3 mL LB broth with 5 μg/mL Erm and 10 μg/mL Cm and incubated at 30 °C for 12–18 h. A 0.5-mL culture was transferred into 50 mL LB containing 10 μg/mL Cm in a 250-mL flask and incubated for 36 h at the same temperature. After then, the temperature was elevated to 42 °C and the culture was incubated for another 24 h with shaking to diminish the plasmid DNA. Finally, the cells were diluted to 10^5^–10^6^ cells/mL with sterile water and dispensed onto the LB plate with 10 μg/mL Cm. The plates were incubated at 42 °C until the colonies were formed. The same colony was picked up and dotted on two LB plates with 10 μg/mL Cm and with 5 μg/mL Erm and 10 μg/mL Cm, respectively. After incubating at 30 °C for about 24 h, the colonies growing only on LB plate with Cm and not on the plate amended with Cm and Erm were picked up for further identification by colony-PCR. The primers (Id-ScoC.F/Id-cm3.R, and Id-cm5.F/Id-scoC.R/) were used to amply the DNA sequences to cover the integrating sites in the genome, which were confirmed by DNA sequencing.

### Growth curve and extracellular protease activity assay

Three *B. pumilus* BA06 strains including *scoC* deletion mutant (BA06-∆scoC), ∆*scoC/scoC*^*+*^ overexpression, and the wt strains were inoculated into 50 mL minimal medium (MM, 1.0 g/L sodium citrate, 2.0 g/L (NH_4_)_2_SO_4_, 14.0 g/L K_2_HP0_4_, 6.0 g/L KH_2_P0_4_, 0.2 g/L MgS0_4_, 2.5 g/L Yeast extract and 5.0 g/L D-glucose) and incubated at 37 °C with shaking at 200 rpm. At the indicated time points, 2 × 1 mL culture was sampled from each flask. The cell density was measured at OD_600_. The extracellular protease activity was determined using casein as substrate by the Folin-Phenol method as described [50]. All the experiments were repeated in triplicate.

### Motility analysis

The motility analysis was performed as described previously [25]. Briefly, one microliter of the freshly overnight culture of each strain grown in LB broth (OD_600_ ~ 1.0) was seeded at the center of the motility assay plates (90-mm in diameter) with a pipette tip. All plates were immediately air-dried at ambient temperatures (~ 37 °C) in a laboratory ventilator. Swimming motility was evaluated on the freshly prepared semi-liquid MM plates containing 0.3% agar. Swarming motility was evaluated on LB plates containing 0.7% agar. Photographs were taken after 24-h incubation at 37 °C. And the diameter of motility halos was measured at the same time. All motility assay experiments were independently repeated more than three times.

### Flagellum staining

Flagellum staining was performed as described by a standard method [51]. Briefly, a loop of bacterial cells was taken from the colony edge growing on MM plate (0.3% agar) at 37 °C for 12 h and dispensed in sterile water on the glass slide. After air-dried, the cells were stained with the solution A (5.0% tannic acid, 1.5 g% FeCl_3_, 0.01% NaOH, 2.0% formalin) for 5 min, subsequently with the solution B (2.0% AgNO_3_) for 0.5 min. After air-dried, the flagella were observed under oil microscope with magnification of × 100 folds. The number of flagella for the cells were counted. The flagellum staining was performed by several times using the various cultures.

### RNA isolation, library construction and transcriptome analysis

The cell samples of *B. pumilus* cultures growing in MM broth were pelleted by centrifugation at 8000 rpm at the indicated time points (12, 24, and 36 h), and then re-suspended in TE buffer (1 mM EDTA and 10 mM Tris-HCl, pH 8.0) supplemented with 1.5 mg/mL lysozyme. After incubated at 37 °C for 10 min, the TRIzol reagent was added. The cell suspension was mixed extensively while using the gauge to disrupt the cells completely. Finally, total RNA was isolated following the instructions provided with the TRIzol reagent. The genomic DNA was removed using the Genome DNA Eraser Kit. The resulting RNAs were fragmented and reverse transcribed using random hexamers as the primer. Second strand cDNA synthesis was performed using DNA Polymerase I and RNase H. The cDNA fragments were processed for end repair and ligated to paired end adaptors. Finally, the library was sequenced on an Illumina HiSeqTM2000.

The clean data of the wt and BA06-∆*scoC* strains growing for 12 h, 24 h and 36 h were mapped to the genomic annotation by HISAT2 software [52, 53], indicating that more than 99% reads could map onto the genome (Additional file 7: Table S4). The expression level of each gene was calculated by StringTie software [53, 54]. The number of reads in features were counted by HTseq [55] or featureCounts [56], which were imported into the edgeR software [57] to calculate differentially expressed genes (DEGs) between various samples and different time points. When the value of |log_2_^fold change^| ≥ 1.3250 and *p* < 0.01, the corresponding gene was defined as the DEG.

Based on the numbers of DEGs that calculated across the three time points of 12 h, 24 h, and 36 h, the Venn diagram was constructed online (http://bioinformatics.psb.ugent.be/webtools/venn/) [58]. KEGG analysis was performed online (www.kegg.jp/kegg/pathway.html) [59]. The matrix hierarchical cluster analysis for the selected KEGG pathways was performed using PermutMatrix v.1.9.3 (www.lirmm.fr/~caraus/PermutMatrix/) [60].

### Quantitative real-time PCR analysis

To confirm the transcriptome data, the quantitative real-time PCR (qPCR) was performed for the selected genes. Totally, 16 pairs of primers were designed (Additional file 8: Table S5) and the 16 sRNA gene was used as the control. The RNAs were isolated from the same cell samples as the transcriptome analysis. The reverse transcription (RT) was performed in 20 μL mixture including 1 μg RNA sample and 5 U RTase (Takara, Dalian, China). The resulting cDNA was diluted by × 10 folds and used as a template for qPCR. qPCR was carried out following the instruction of 2 × Real Time PCR EasyTM-SYBR Green I Kit. The calculated cycle threshold (CT) was normalized to the CT of 16 sRNA amplified from the corresponding sample. Changes in mRNA levels were calculated using the 2^-ΔΔCT^ method [61].

### Statistical analysis

Statistical analysis was performed with SPSS Statistics software. A *P* value of 0.05 or 0.01 was considered significant or extreme significant.

## Additional files

Additional file 1: **Figure S1.** Strategy of *scoC* disruption (A) and identification of the *scoC* deletion mutant by colony-PCR and DNA sequencing. B, left integrating site; C, right integrating site. (JPG 269 kb)

Additional file 2: **Figure S2.** Formation of endospore (A) and biofilm (B) of *scoC* mutant (BA06-∆*scoC*), the wt (BA06) and overexpression (∆*scoC/scoC*^+^) strains of *B. pumilus*. (PNG 355 kb)

Additional file 3: **Table S1.** The relative expression levels and the fold-change of all the genes in *scoC* mutant (BA06-∆*scoC*) and the wt (BA06) strains of *B. pumilus* across three time points. (XLSX 2020 kb)

Additional file 4: **Figure S3.** The organization of flagella-related genes in *B. pumilus* BA06 genome and their fold-change of transcription at 12 h between the wt and *scoC* mutant strains. The arrow indicates the putative transcriptional direction. (PNG 61 kb)

Additional file 5: **Table S2.** Confirmation of the transcriptional data for the selected genes by qPCR. (DOCX 17 kb)

Additional file 6: **Table S3.** The primers used to construct the vectors and for DNA sequencing. (DOCX 16 kb)

Additional file 7: **Table S4.** Overall mapped reads generated by RNA-seq onto the genome of *B. pumilus* BA06. (DOCX 17 kb)

Additional file 8: **Table S5.** The primers used in qPCR to confirm the transcriptome data for the selected genes. (DOCX 18 kb)

## Abbreviations

Amp: Ampicillin
Cm: Chloramphenicol
DEG: Differentially expressed gene
Erm: Erythromycin
Kan: Kanamycin
LB: Luria-Bertani
PCR: Polymerase chain reaction
qPCR: Quantitative real-time PCR
RT: Reverse transcription
wt: Wild-type
