# Supplementary figures and images for: Disruption of the pleiotropic gene scoC causes transcriptomic and phenotypical changes in Bacillus pumilus BA06
Source: BMC Genomics. 2019 Apr 30;20:327. doi: 10.1186/s12864-019-5671-8 (PMC6492404; doi:10.1186/s12864-019-5671-8)

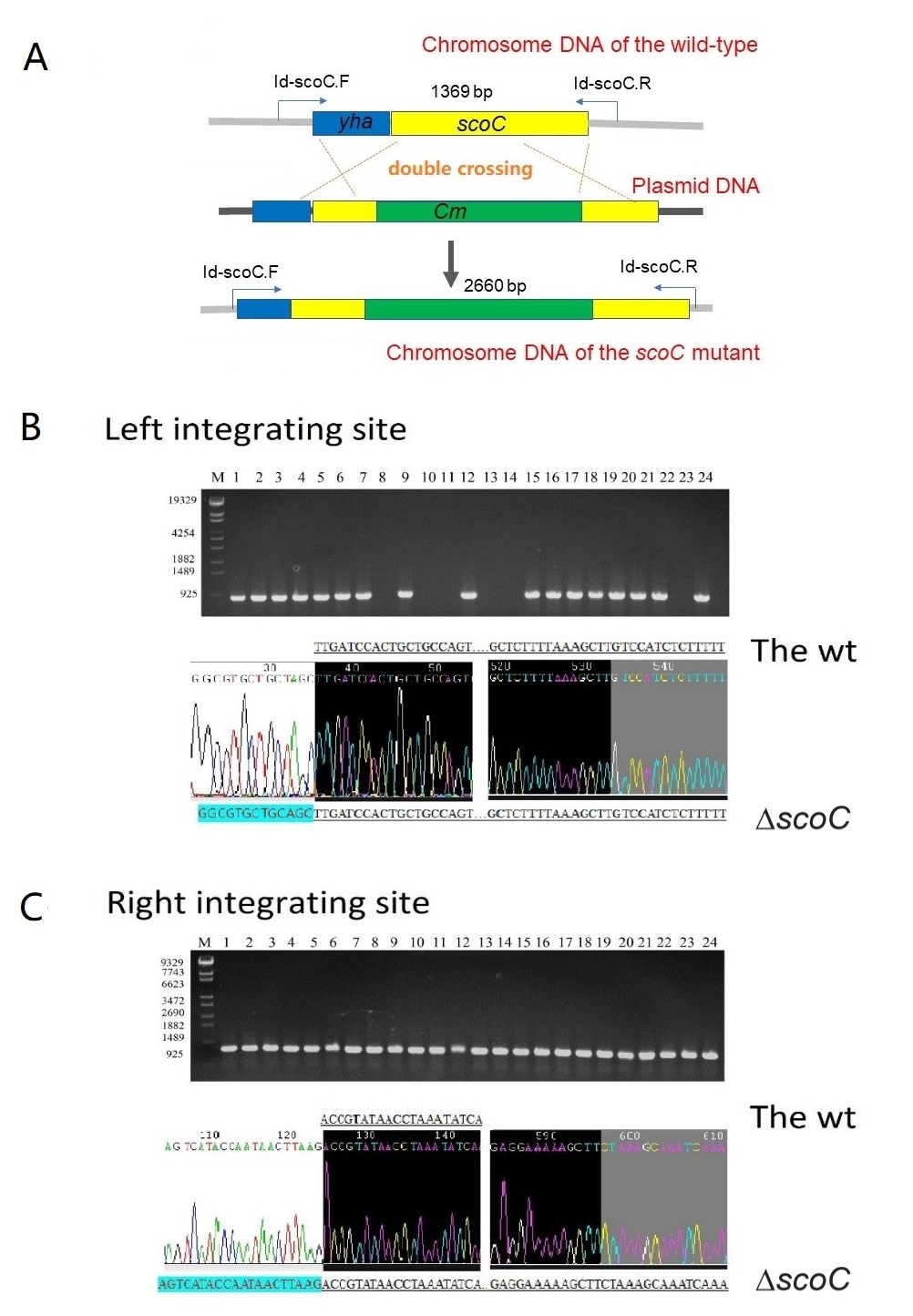

Supplement: Supplementary file 1 — Figure S1. Strategy of scoC disruption (A) and identification of the scoC deletion mutant by colony-PCR and DNA sequencing. B, left integrating site; C, right integrating site. (JPG 269 kb) [file 12864_2019_5671_MOESM1_ESM.jpg]

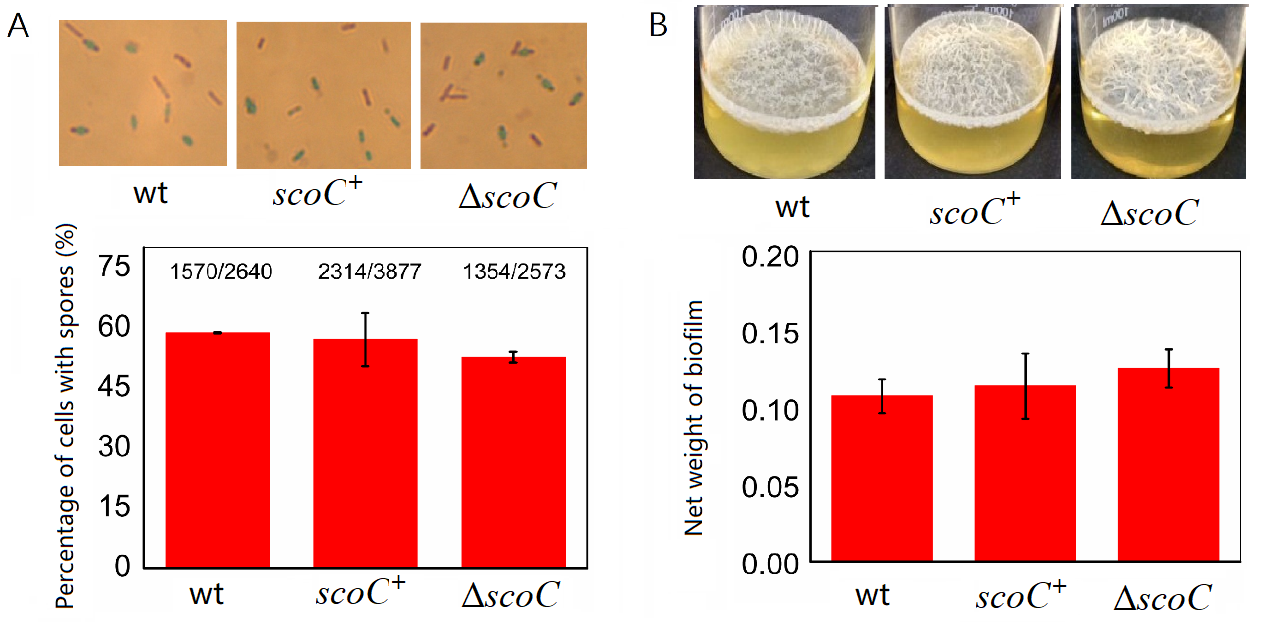

Supplement: Supplementary file 2 — Figure S2. Formation of endospore (A) and biofilm (B) of scoC mutant (BA06-∆scoC), the wt (BA06) and overexpression (∆scoC/scoC+) strains of B. pumilus. (PNG 355 kb) [file 12864_2019_5671_MOESM2_ESM.png]

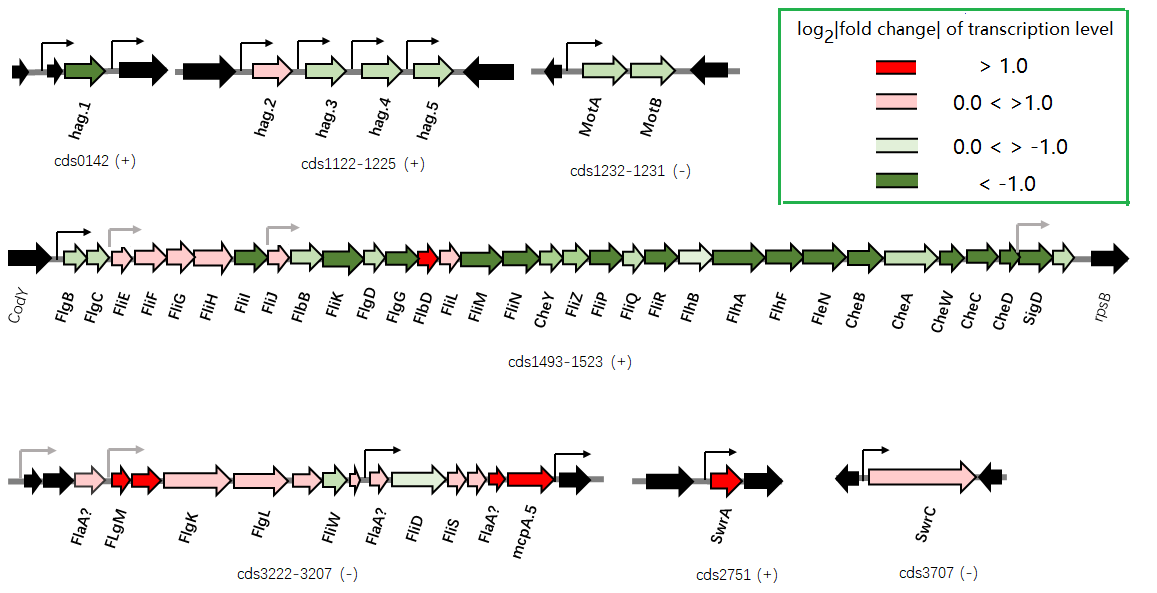

Supplement: Supplementary file 4 — Figure S3. The organization of flagella-related genes in B. pumilus BA06 genome and their fold-change of transcription at 12 h between the wt and scoC mutant strains. The arrow indicates the putative transcriptional direction. (PNG 61 kb) [file 12864_2019_5671_MOESM4_ESM.png]
