# Supplementary material for: Disruption of the pleiotropic gene scoC causes transcriptomic and phenotypical changes in Bacillus pumilus BA06
Source: BMC Genomics. 2019 Apr 30;20:327. doi: 10.1186/s12864-019-5671-8 (PMC6492404; doi:10.1186/s12864-019-5671-8)
Supplement: Supplementary file 5 — Table S2. Confirmation of the transcriptional data for the selected genes by qPCR. (DOCX 17 kb) [file 12864_2019_5671_MOESM5_ESM.docx]

**Table S2** Confirmation of the transcriptional data for the selected genes by qPCR

| **Gene ID** | **Name** | **Log _2_ (fold-change) by qPCR** | | | **Log _2_ (fold-change) by RNA-seq** | | |
| --- | --- | --- | --- | --- | --- | --- | --- |
|  |  | **12 h** | **24 h** | **36 h** | **12 h** | **24 h** | **36 h** |
| cds0935 | *aprE* | 3.0620 | 2.3363 | 1.5597 | 1.6123 | 3.5830 | 1.8637 |
| cds2061 | *aprN* | -0.7707 | -2.2457 | -2.2961 | -1.9167 | -1.8044 | -2.1735 |
| cds3474 | *vpr* | -0.3464 | -1.0528 | -0.4354 | -0.1194 | -0.7412 | -0.9345 |
| cds0014 | *abrB* | 0.4985 | -0.3476 | 1.0587 | 0.1661 | -1.2795 | 0.6369 |
| cds3225 | *degU* | 0.1552 | -0.0245 | -0.5482 | -0.2210 | -0.0270 | -0.4446 |
| cds1125 | *hag5* | -0.6904 | -1.9185 | -2.0821 | -0.8634 | -1.9421 | -1.4039 |
| cds1231 | *motB* | -0.3546 | -1.0865 | -1.9607 | -0.8541 | -2.4570 | -1.1367 |
| cds1256 | *mcpC* | -0.3110 | -0.1177 | 0.9368 | -0.4942 | -1.0152 | -1.4362 |
| cds1508 | *fliN* | -1.2055 | -0.7186 | -2.3558 | -2.2621 | -0.5208 | -1.1605 |
| cds1514 | *flhB* | -0.5667 | -1.5324 | -3.8796 | -0.0362 | -1.4807 | -1.4000 |
| cds1518 | *cheB* | -0.8366 | -1.8686 | -2.3925 | -1.8342 | -2.2539 | -1.3681 |
| cds1522 | *cheD* | -0.8182 | -2.1249 | -2.3673 | -1.2692 | -1.3174 | -0.7076 |
| cds1523 | *sigD* | -0.6829 | -1.2006 | -1.9063 | -1.3372 | -2.0092 | -1.0209 |
