# Supplementary material for: Disruption of the pleiotropic gene scoC causes transcriptomic and phenotypical changes in Bacillus pumilus BA06
Source: BMC Genomics. 2019 Apr 30;20:327. doi: 10.1186/s12864-019-5671-8 (PMC6492404; doi:10.1186/s12864-019-5671-8)
Supplement: Supplementary file 6 — Table S3. The primers used to construct the vectors and for DNA sequencing. (DOCX 16 kb) [file 12864_2019_5671_MOESM6_ESM.docx]

**Table S3** The primers used to construct the vectors in this work and for DNA sequencing

| **Primers** | **Sequences (5’-3’)** | **Remarks** |
| --- | --- | --- |
| B6sco.F | TATGGTATTTGTCTTCCCGATTGC |  |
| B6sco.R | GTGTGCGTACATTGGAAATGCC |  |
| Sco-Afl.F | ATTATTgctagcTTGATCCACTGCTGCCAGTCC | Afl II |
| Sco-Nhe.R | TATTcttaagACCGTATAACCTAAATATCAATGAAC | Nhe I |
| B06Pgpd.F | agatatcatatGCAGGCGGCTTTTGACCGCC | Nde I |
| B06Pgpd.R | GTGGTGGTGGTGGTGGTGCATGGCTAACGACCCTTTCTTT | Overlapped PCR |
| B06P-scoC.F | ATGCACCACCACCACCACCACAATCATTCGGAACAGCCTTTCG | Overlapped PCR |
| B06P-scoC.R | gggcccCTCGAGTTAAACAATTTGATTTGCTTTAGAAG | Xho I |
| pSU03ΔAp.F | cagatatctcgAGCTTCTAACTAAAATAAGTGTGAC | Xho I |
| pSU03ΔAp.R | taatctCATATGTAATCGTCGACCTGCAGGCATGC | Nde I |
| Id-scoC.F | AGAGGCAAAGGACATTGAGCG | Identifying mutant |
| Id-scoC.R | GATTGGAATGGCTAGTGGACG | Identifying mutant |
| Id-cm5.F | ATAAGGGTAACTATTGCCGTCG | For sequencing |
| Id-cm3.R | ATTCAGGAATTGTCAGATAGGC | For sequencing |
