# Supplementary material for: Disruption of the pleiotropic gene scoC causes transcriptomic and phenotypical changes in Bacillus pumilus BA06
Source: BMC Genomics. 2019 Apr 30;20:327. doi: 10.1186/s12864-019-5671-8 (PMC6492404; doi:10.1186/s12864-019-5671-8)
Supplement: Supplementary file 7 — Table S4. Overall mapped reads generated by RNA-seq onto the genome of B. pumilus BA06. (DOCX 17 kb) [file 12864_2019_5671_MOESM7_ESM.docx]

**Table S4** Overall mapped reads generated by RNA-seq onto the genome of *B. pumilus* BA06

| **Sample name** | **Left**  **size (G)** | **Right**  **size (G)** | **Number of**  **clean reads** | **Mapped reads**  **(%)** | **Paired reads**  **(%)** | **GC**  **(%)** |
| --- | --- | --- | --- | --- | --- | --- |
| wt-12h | 4.17 | 4.17 | 25,876,024 | 25,814,985 (99.76) | 25,876,024 (100) | 42 |
| ∆*scoC*-12h | 2.89 | 2.89 | 17,928,228 | 17,800,021 (99.28) | 17,928,228 (100) | 40 |
| wt-24h | 3.94 | 3.94 | 24,432,610 | 24,354,424 (99.68) | 24,432,610 (100) | 40 |
| ∆*scoC*-24h | 2.93 | 3.93 | 24,406,906 | 24,307,397 (99.59) | 24,406,906 (100) | 40 |
| wt-36h | 3.95 | 3.95 | 24,474,136 | 24,404,177 (99.71) | 24,474,136 (100) | 42 |
| ∆*scoC*-36h | 4.00 | 4.00 | 24,836,224 | 24,739,745 (99.61) | 24,836,224 (100) | 42 |
