# Supplementary material for: Disruption of the pleiotropic gene scoC causes transcriptomic and phenotypical changes in Bacillus pumilus BA06
Source: BMC Genomics. 2019 Apr 30;20:327. doi: 10.1186/s12864-019-5671-8 (PMC6492404; doi:10.1186/s12864-019-5671-8)
Supplement: Supplementary file 8 — Table S5. The primers used in qPCR to confirm the transcriptome data for the selected genes. (DOCX 18 kb) [file 12864_2019_5671_MOESM8_ESM.docx]

**Table S5** The primers used in qPCR to confirm the transcriptome data for the selected genes

| **Gene ID** | **Gene name** | **Primer name** | **Upstream primer (5’-3’)** | **Downstream primer (5’-3’)** |
| --- | --- | --- | --- | --- |
|  | 16sRNA | 16sRNA.F/R | AGCCGCGGTAATACGTAGG | TCCACTCTCCTCTTCTGCAC |
| cds00935 | *aprE* | q0935.F/R | TAGCTTCGTCCCTTCAGAGCC | CTTTAACGGCATACAAGGAGGC |
| cds02061 | *aprN* | q2061.F/R | GTTCCAACAGAGTCCGATCCG | TGGTGCCACACCAACGACGC |
| cds03225 | *degU* | q3225.F/R | CACTATCATCCGGATGTTGTC | GTGTCACATAGTTTTCATCGTC |
| cds00014 | *abrB* | q0014.F/R | TCGCCGTACGTTAGGAATCGC | GTTATCGTCTGATACTTCACCTG |
| cds02132 | *spo0A* | q2132.F/R | AAGAGCCCGACGTTCTCCTC | CGTCAGCATAATGACACTTGGC |
| cds01125 | *hag* | q1125.F/R | GTGCAGGAGATGACGCAGCG | TAAGAGAAATACCATCTTGTGCG |
| cds01508 | *fliN* | q1508.F/R | CTGCTCCTATGCAGCAATCGG | CTGACTTGTTGTATGCTGAGGC |
| cds01514 | *flhB* | q1514.F/R | GGGTCTATATGCAGCAGGTGC | TTGATGTCTTGTTTAGACATCCG |
| cds01518 | *cheB* | q1518.F/R | GCTTTAGTGATCAGGCTGTCC | CATTCCTGTCATAATGACAGCG |
| cds01522 | *cheD* | q1522.F/R | GCTAAGTATGCAGATACAGGTG | TTCTCATCAGATCATTGGTTGAC |
| cds01523 | *sigD* | q1523.F/R | TGTGACACCAACAGAGGTTGC | CCGTCTTCTTGATCATGCAGC |
| cds01231 | *motB* | q1231.F/R | CAAAGCTGACGGATGAAGGTC | TGGCTAACGGAACATCCTGCG |
| cds01256 | *mcpC* | q1256.F/R | TTCTCATCAGATCATTGGTTGAC | ATTCTGCAGATTGTTCAGCCAG |
